# The role of microvesicles containing microRNAs in vascular endothelial dysfunction

**DOI:** 10.1111/jcmm.14716

**Published:** 2019-10-01

**Authors:** Zeyu Shu, Jin Tan, Yuyang Miao, Qiang Zhang

**Affiliations:** ^1^ Department of Geriatrics Tianjin Medical University General Hospital Tianjin Geriatrics Institute Tianjin China; ^2^ Tianjin Medical University Tianjin China

**Keywords:** atherosclerosis, endothelial dysfunction, inflammation, microRNA, microvesicle

## Abstract

Many studies have shown that endothelial dysfunction is associated with a variety of cardiovascular diseases. The endothelium is one of the primary targets of circulating microvesicles. Besides, microRNAs emerge as important regulators of endothelial cell function. As a delivery system of microRNAs, microvesicles play an active and important role in regulating vascular endothelial function. In recent years, some studies have shown that microvesicles containing microRNAs regulate the pathophysiological changes in vascular endothelium, such as cell apoptosis, proliferation, migration and inflammation. These studies have provided some clues for the possible roles of microvesicles and microRNAs in vascular endothelial dysfunction‐associated diseases, and opened the door towards discovering potential novel therapeutic targets. In this review, we provide an overview of the main characteristics of microvesicles and microRNAs, summarizing their potential role and mechanism in endothelial dysfunction, and discussing the clinical application and existing problems of microvesicles for better translational applications.

## 1 INTRODUCTION

In the 1950s, the innovative studies of Palade and Gowan described the endothelium as a dynamic organ with diverse capabilities for the first time.^1^ Over the last decades, besides as a vast, selectively permeable interface separating the vascular and interstitial compartments of the body,^2^ the endothelium has been widely investigated as an active organ secreting numerous mediators, which are necessary for normal vascular function.^3^, ^4^ Endothelial dysfunction is characterized by shifting of the physiological balance of the vessel towards a vasoconstrictive, pro‐thrombotic and pro‐inflammatory state,^5^ often preceding atheroma development and being linked to vasculopathic diseases such as acute coronary syndromes (ACSs), coronary artery disease (CAD), hypertension, diabetes mellitus (DM), stroke and peripheral arterial disease.^6^, ^7^, ^8^, ^9^, ^10^ The endothelium is one of the primary targets of circulating microvesicles (MVs).^11^ In recent years, microRNAs (miRNAs) have emerged as an important regulatory factor for the function of endothelial cells (ECs) by fine‐tuning gene expression.^12^ MVs operate as a delivery system of miRNAs, playing an active and important role in regulating vascular endothelial function. The study of MVs performing as miRNA carriers highlights the association of MVs in health and disease condition. Thus, the studies of MVs and associated miRNAs in the cycle may deepen the understanding of endothelial dysfunction and related diseases. This review summarizes the novel findings of the role and potential mechanism of MVs and their associated miRNAs in endothelial dysfunction.

## 2 OVERVIEW OF MVs AND miRNAs

### 2.1 MVs

Extracellular vesicles (EVs) are a heterogeneous population of particles, which are released from various cell types into the extracellular space.^13^ According to their size, biogenesis and secretion mechanisms, EVs can be categorized as exosomes, MVs (also known as microparticles) and apoptotic bodies.^14^, ^15^, ^16^ MVs are irregularly shaped submicron vesicles (100‐1000 nm)^17^ released from many types of cells, including platelets, ECs, erythrocytes and leucocytes.^18^, ^19^ Moreover, MVs have been found in blood, urine, synovial fluid, extracellular spaces of solid organs, atherosclerotic plaques, tumours and elsewhere.^20^ Long considered as inert debris,^21^ MVs are now appreciated as an important transcellular delivery system in the exchange of biological signals,^22^ and their release is the result of a highly regulated process.^23^

**Figure 1:**
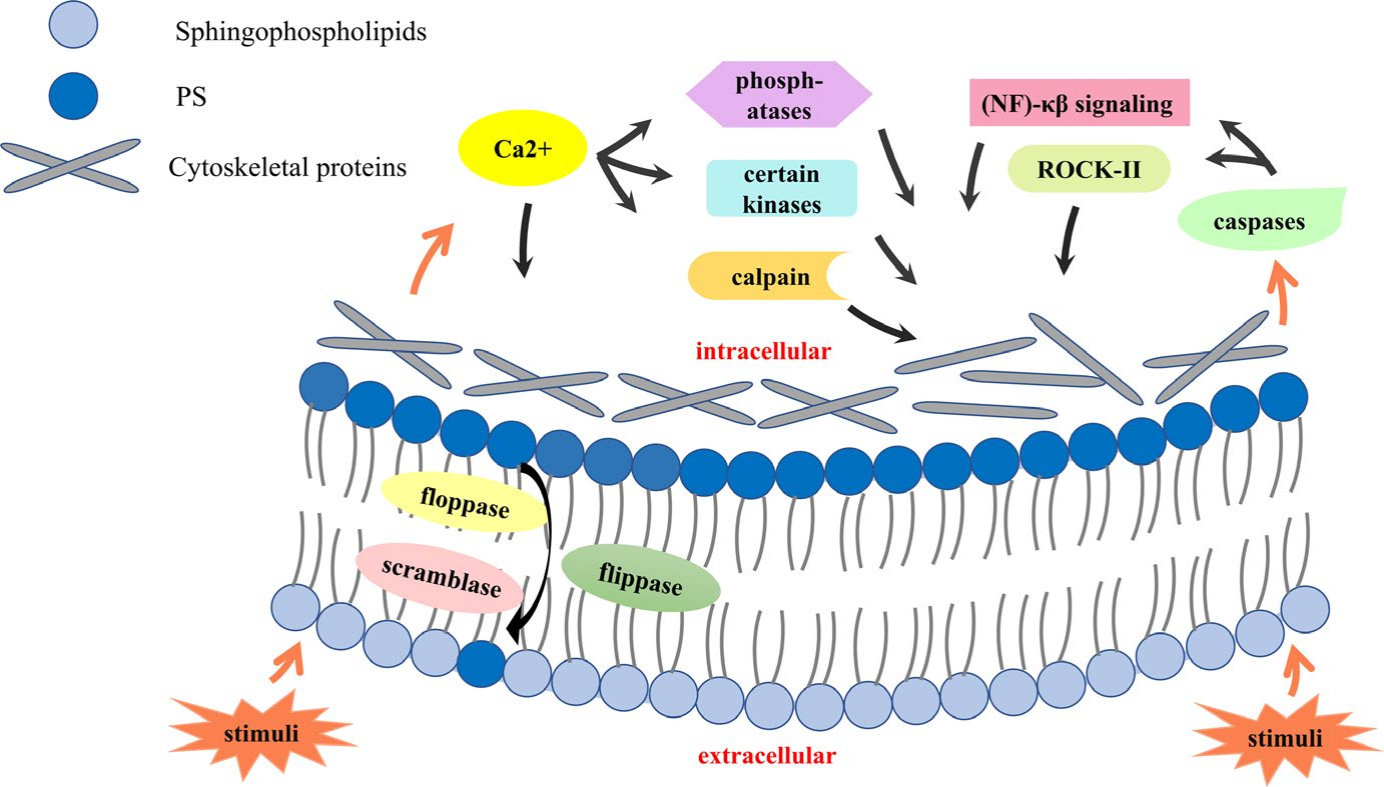
The process of MV formation and release from cells. External stimuli followed by increasing intracellular Ca2+ that inhibits the flippase and activates floppase and scramblase with PS movement from the inner face to the outer face of cell membrane. Besides, the increased intracellular Ca2+ activates calpain and certain kinases and inhibits phosphatases, generating the cytoskeleton reorganization and/or damage, facilitating MV release. Under certain stimuli, ROCK‐II activation by caspase‐2 and the involvement of nuclear factor (NF)‐κβ signalling can lead to cytoskeleton reorganization. PS: phosphatidylserine; ROCK‐II: Rho‐associated protein kinase 2

The formation and release of MVs are the result of a complex process with cytoskeleton reorganization and loss of the physiological asymmetry of the membrane bilayer.^24^ The formation of MVs appears to occur mostly in lipid‐rich microdomains (lipid rafts/caveolae) within the plasma membrane.^25^ The underlying mechanism of the formation and release of MVs remains to be fully elucidated, but a consensus has been reached that intracellular Ca2+ plays a crucial role in the formation and release of MVs^26^ (Figure 1). Firstly, physiological asymmetry of the membrane bilayer is maintained by several phospholipid transporters: flippase, floppase and scramblase. Under physiological conditions, phosphatidylserine (PS) and phosphatidylethanolamine (PE) are continuously internalized due to the role of flippase, while floppase translocates them to the outside. Scramblase helps promote non‐specific bidirectional redistribution across the bilayer.^27^ In the presence of external stimuli, increasing intracellular Ca2+ inhibits the flippase^28^ and activates floppase and scramblase with role in the PS movement from the inner monolayer of the plasma membrane to the surface of MVs.^29^, ^30^ Secondly, the increasing intracellular Ca2+ leads to calpain activation and thus generates the cytoskeleton reorganization and/or damage.^31^ Lastly, intracellular Ca2+ activates certain kinases and inhibits phosphatases to ensure the cleavage of the cytoskeleton and facilitate MV release.^32^ Besides, ROCK‐II activated by caspase‐2 and the involvement of nuclear factor (NF)‐κβ signalling can lead to cytoskeleton reorganization.^33^ Rho‐associated kinase (ROCK‐I) has been proven to be relevant to the shedding of apoptotic MVs.^34^

The formation and release of MVs are related to a wide range of stimuli, such as pro‐inflammatory cytokines (eg tumour necrosis factor‐α and interleukin‐1β), thrombin, complement proteins (C5b‐9), uraemic toxins and reactive oxygen species.^35^, ^36^ This makes MVs well suited as surrogate marker of endothelial dysfunction.

### 2.2 miRNAs

miRNAs are a class of single‐stranded, small (about 22 nucleotides long) and generally non‐coding RNAs, which have emerged as critical regulators of gene expression via post‐transcriptional degradation or translational repression.^37^, ^38^, ^39^ Each mRNA molecule may be regulated by multiple miRNAs, and a single miRNA may influence the expression of a wild range of genes in a cell.^40^ Some miRNAs are expressed ubiquitously, and some miRNAs are expressed in a tissue‐specific and/or stage‐specific manner.^41^, ^42^ It has been estimated that miRNAs regulate the activity of 30%‐50% of protein‐coding genes^43^ and modulate about 10%‐30% of human genome expression.^44^ miRNAs play a role in regulating various biological processes including metabolism, cell proliferation and differentiation, and apoptosis.^45^, ^46^, ^47^ The function of miRNA is directly associated with structure; thus, analysing the process of miRNA biogenesis may provide new ideas for the study of miRNA.

**Figure 2:**
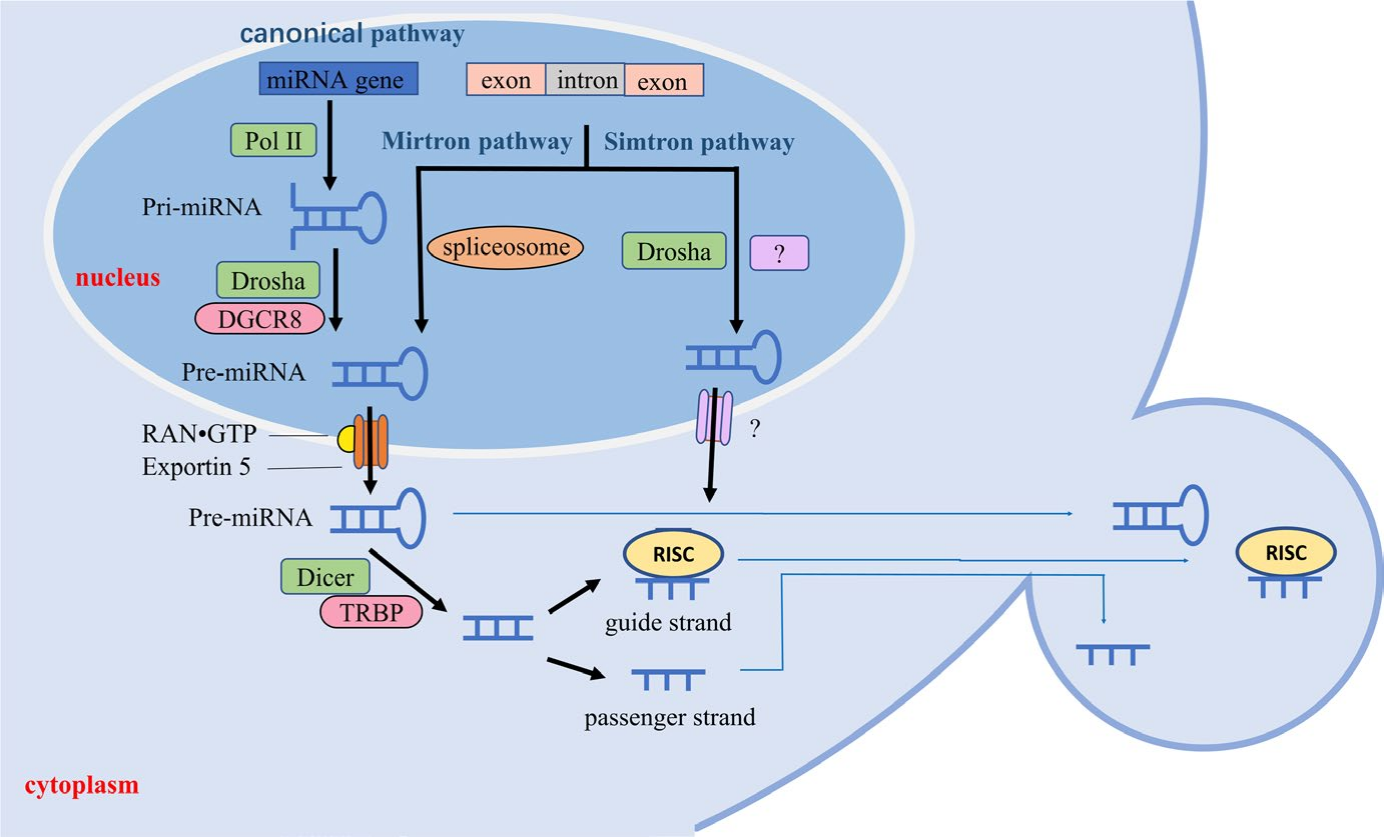
The formation of miRNA. In the canonical pathway, a miRNA gene is transcribed by RNA Pol II to generate pri‐miRNA, which is subsequently cleaved by Drosha along with DGCR8 into pre‐miRNA. In the mirtron pathway, mirtrons are excised from the host gene by spliceosome and trimming of short introns without Drosha processing. Pre‐miRNAs generated by both pathways are transported to the cytoplasm by exportin5‐RAN•GTP complex, further cleaved by the RNase III Dicer and TRBP to double‐stranded miRNA. Guide strand is preferentially incorporated in the RISC. In the simtron pathway, Drosha and possibly an unknown conjugate are involved in simtron biogenesis. After further processed by unknown factors, simtron enters the RISC complex. Functional miRNAs are produced by all three pathways. Most miRNAs are localized intracellularly, but some of them are released into the blood by being packaged in MVs. Both miRNA (including guide strand and passenger strand) and pre‐miRNA can be packaged into MV to deliver information. Pol II:RNA polymerase II; Drosha:RNase III endonuclease; RAN•GTP:GTPase Ran; DGCR8: Di George syndrome critical region 8; TRBP:TAR RNA‐binding protein; RISC: RNA‐induced silencing complex. The places labelled with question marks are proposed but not clear

The generation of mature miRNA is a complicated process (Figure 2). Within the nucleus, a miRNA gene is transcribed by RNA polymerase II (RNA Pol II) to generate pri‐miRNA, which is subsequently cleaved by RNase III endonuclease Drosha along with the cofactor protein DGCR8 into pre‐miRNA with a hairpin structure. The pre‐miRNA is transported to the cytoplasm by exportin5‐RAN•GTP complex, and is further cleaved by the RNase III Dicer and TAR RNA‐binding protein (TRBP) to ~22‐nucleotide double‐stranded miRNA.^48^, ^49^ One strand, called a guide strand, is incorporated in the RNA‐induced silencing complex (RISC),^50^ while the other one, called a passenger strand, is typically degraded or incorporated into MVs and released from the cell, and therefore less abundant than the guide strand.^51^ However, Schober et al detected appreciable expression levels of the miR‐126‐5p passenger strand, which promoted endothelial repair.^52^ This shows that the passenger strand can play a role instead of being degraded. Alternatively, some atypical miRNAs, such as mirtrons and simtrons, can be generated by non‐canonical pathways. Mirtrons can be processed independently of the Drosha through the direct splicing of introns. Simtrons are splicing‐independent mirtron‐like miRNAs, which processing involves Drosha and possibly an unknown binding partner but does not require DGCR8, Dicer, Ago2 or exportin5.^53^, ^54^

Most miRNAs are localized intracellularly, but some of them are released into the blood by combining with proteins or being packaged in EVs.^51^ It has long been reported that miRNAs are a significant cargo contained by MVs.^55^ Advances in this field will contribute to a better understanding of the role of delivered miRNAs in pathology and enable them to be identified as therapeutic targets.^56^

## 3 miRNAs ARE FOUND IN MVs AND TRANSFERRED BETWEEN CELLS

The original idea of EV transfer of miRNAs began in 2007 when a seminal study by Valadi et al proved exosomes to contain functional miRNAs, which can be delivered to another cell.^57^ In addition, many studies have reported that functional miRNAs transferred by MVs mediate cell‐cell communication.^58^, ^59^ Intercellular communication processes based on extracellular miRNAs can be considered as consisting of 3 key steps: (a) miRNAs are secreted from cells and selectively packaged into appropriate carriers. (b) miRNAs are protected from circulating RNases and transferred to targeted cells. (c) miRNAs retain the ability to recognize and repress mRNA targets within recipient cells.^60^

### 3.1 miRNAs are selectively encoded into appropriate carriers

Firstly, only selected part of the intracellular miRNAs are effectively transferred to recipient cells.^61^, ^62^, ^63^ miRNAs must be actively secreted from cells and selectively packaged into appropriate carriers, such as EVs (exosomes, MVs and apoptosis bodies), lipoproteins (LDL, HDL) and other ribonucleoprotein complexes.^64^, ^65^ Selective and precise transport of miRNAs by MVs is the key step of extracellular miRNA‐mediated intercellular communication.^66^, ^67^ Multiple miRNAs can be incorporated into one MV, but the exact mechanism of packaging is unclear.^68^ Changes in miRNA content between MVs and their parental cells indicate a selective packaging of miRNAs into MVs.^69^

In some cases, precursors of mature miRNAs are also released.^70^ Depending on Rho A/ROCK signalling, both precursor and mature forms of miRNAs are exported into MVs in response to TNF‐α^71^ (Figure 1). Besides, not all of the miRNAs in the MVs are associated with the Ago2, the major component of the RISC.^72^ For example, miR‐126‐5p passenger strand, which does not bind to the Ago2, can exist and promote endothelial repair.^52^

### 3.2 miRNAs are protected from circulating RNases and transferred to recipient cells

Secondly, miRNAs are protected from circulating RNases and transferred to recipient cells. Both MVs and proteins contribute to protecting miRNAs in the MVs from RNases even in the unfavourable physiological condition. MV’s lipid bilayers contribute to maintaining the stability of the circulating miRNAs to ensure the miRNA transfer to the recipient cells.^73^, ^74^ The protection by MVs is non‐specific, whereas by proteins is specific.^72^ Arroyo et al and Turchinovich et al demonstrated that the MV‐free miRNAs were protected from RNase A by the association with Ago2 complexes after the disruption of the MVs.^75^, ^76^ Interestingly, not all of the miRNAs in the MVs are associated with Ago2 complexes, and different miRNAs are associated with Ago2 complexes to different degrees. Therefore, the protective effect of Ago2 complex on various miRNAs in MVs is different.^72^ The protective effect of Ago2 complexes or other proteins on the extracellular miRNAs in the MVs has not been clear.

Interaction between MVs and recipient cells operates through two main mechanisms: (a) receptor‐ligand interaction— interaction between specific ligands on MVs surface and receptors on target cells leads to subsequent cascade responses; and (b) direct transferring part of their content or component to target cells.^31^, ^77^ When MVs interact with target cells, perhaps the two mechanisms can influence each other. For instance, surface molecules not only act as adhesion molecules to promote receptor‐ligand interaction, but also may act as signalling molecules to regulate the release of the MV content.^78^ Furthermore, a study showed that miRNA‐rich MVs and miRNA‐poor MVs had different ability to be taken up by recipient cells, but this study did not discuss the mechanisms underlying this increase in MV uptake.^71^

### 3.3 miRNAs recognize and repress mRNA targets within recipient cells

Thirdly, miRNAs must retain the ability of recognition and repression of mRNA targets within recipient cells. In cells, miRNAs recognize the specific binding sites usually located in the 3′ untranslated region (3′ UTR) of target mRNA sequences, leading to the reduction of protein expression by inhibiting mRNA translation and/or promoting target mRNA degradation.^79^ Interestingly, recent studies have found that miRNAs may also modulate gene expression in a positive manner.^80^ For instance, Orom UA et al demonstrated that miR‐10a could bind to the 5′ UTR of ribosomal protein mRNAs and enhanced their translation.^81^

Ago2 is also a key effector of miRNA function. Some results may imply that only the secreted miRNAs associated with Ago2 complexes in the MVs are stable and have biological function, compared with the non‐Ago2 complex‐bound miRNAs, which may be simply degraded in the recipient cells.^72^ However, we cannot exclude the possibility that non‐Ago2 complex‐bound miRNAs in the MVs may integrate the miRNA machinery of ECs to mediate their mRNA regulatory effects.^82^ Besides, Alexy et al demonstrated that among three precursor miRNAs assessed, pre‐miR‐155 was released into MVs most consistently in response to TNF‐α, and miR‐155 appeared to have the greatest capacity to be stably transferred to recipient cells. Perhaps extracellular miRNA‐mediated target gene suppression requires transfer of pre‐miRNA, which has a greater capacity to be incorporated into the RISC of recipient cells than mature miRNA.^71^

## 4 THE ROLE OF MVs CONTAINING SPECIFIC miRNAs IN VASCULAR ENDOTHELIAL DYSFUNCTION

### 4.1 Platelet‐derived microvesicles and their miRNA cargo

Platelets have been shown to contain an abundant and diverse array of miRNAs,^83^, ^84^ and two‐thirds of peripheral blood MVs are likely derived from platelets.^85^ Platelets do not have nucleus and cannot undergo miRNA generation within the nucleus, so Figure 2 may not be appropriate for platelets. Some members of the cytoplasmic miRNA processing complex such as Dicer, Ago2 and TRBP2 have been reported in platelets, but platelets inherit most of their mature miRNAs directly from the megakaryocytes.^86^ In addition, because prevalence of the platelet‐derived microvesicles (PMVs) elevates in cardiovascular diseases,^22^ the interaction between miRNAs in PMVs and the endothelial function has always been a focus of attention.

In 2013, Gidlof et al used RNA‐seq to confirm that 9 miRNAs were differentially expressed in PMVs in patients with myocardial infarction. Transfer of miR‐320b by PMVs resulted in a down‐regulation of intercellular adhesion molecule‐1 (ICAM‐1) expression in the HMEC‐1 cells (human microvascular endothelial cell line) and was attenuated in the presence of brefeldin A, an inhibitor of vesicle formation.^87^ This finding is one of the first reports of vesicle‐mediated platelet miRNA transfer, suggesting that transfer of functional platelet miRNAs into vascular ECs via PMVs can play a key role in regulating the inflammatory response of ECs.

In hypertensive conditions, PMVs delivered miR‐142‐3p into ECs and then enhanced abnormal proliferation of ECs by acting on Bcl‐2‐associated transcription factor 1 (BCLAF1). These results indicate that PMVs transmit miR‐142‐3p from activated platelets into ECs and that miR‐142‐3p may play a crucial role in EC dysfunction.^88^ Anti‐β2‐glycoprotein I (anti‐β2GPI) antibodies are the most common antiphospholipid antibodies in antiphospholipid syndrome (APS). Compared with the control group, anti‐β2GPI/β2GPI complex induces the release of PMVs containing higher amounts of miR‐96 and miR‐26a. These two kinds of miRNAs can inhibit migration and tube formation of HUVECs by targeting selectin‐P (SELP) and platelet‐derived growth factor receptor alpha (PDGFRα) to enhance vascular endothelial cell damage.^89^ Recently, a data showed for the first time that miR‐let‐7a delivered by PMV targeted the 3′ UTR of anti‐angiogenic thrombospondin‐1 (THBS‐1) mRNA and potently inhibited THBS‐1 protein synthesis to drive endothelial tubule formation in vitro.^56^

**Figure 3:**
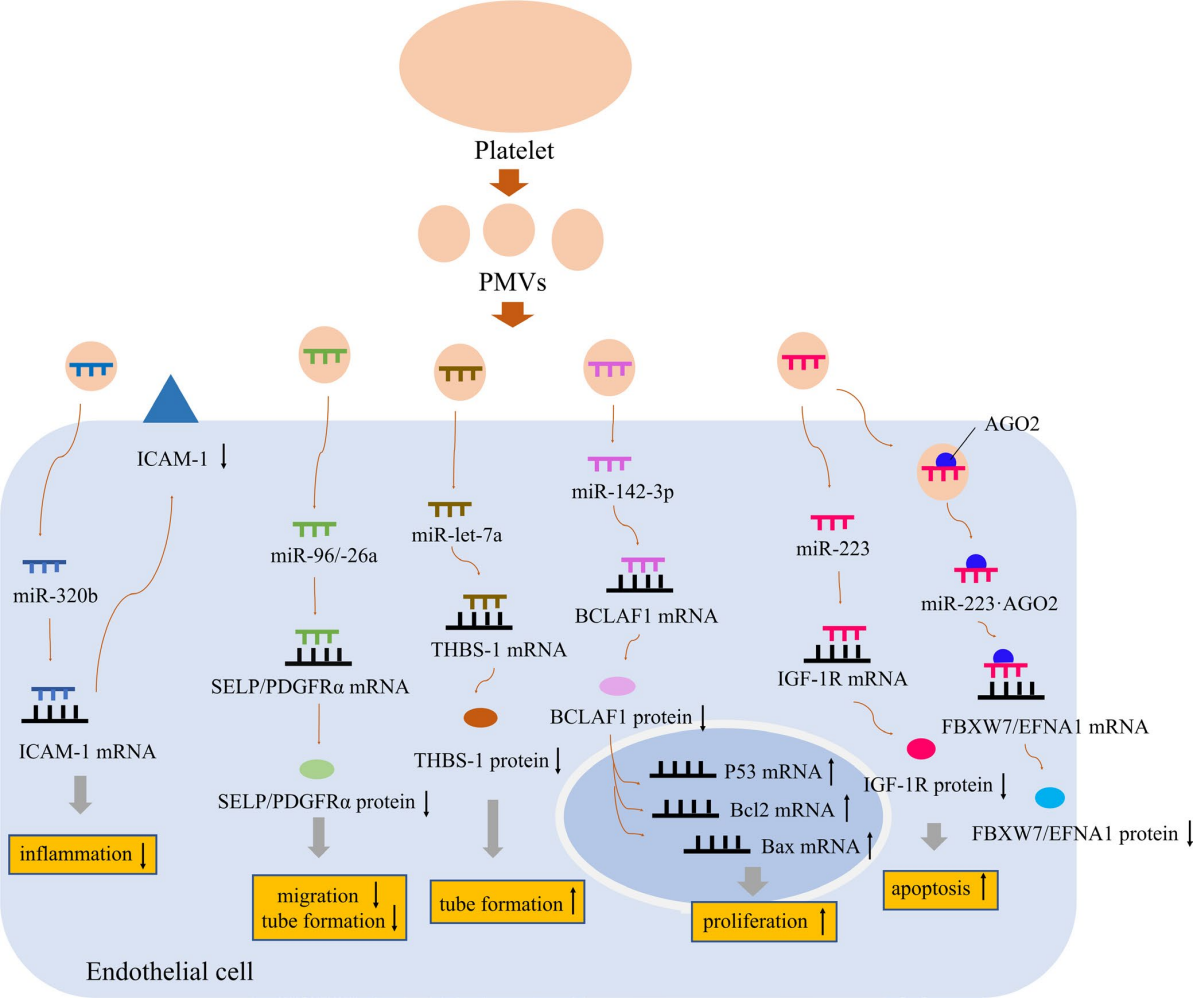
The role of PMVs containing specific miRNAs in vascular endothelial dysfunction. PMVs produced by platelets under different stimuli contain different contents. Different miRNAs in PMVs act on corresponding target genes to affect gene expression and therefore affect vascular endothelial function, including inflammation, cell proliferation and apoptosis. PMVs: platelet‐derived microvesicles; ICAM‐1: intercellular adhesion molecule‐1; SELP: selectin‐P; PDGFRA: platelet‐derived growth factor receptor alpha; THBS‐1: thrombospondin‐1; BCLAF1:Bcl‐2‐associated transcription factor 1; IGF‐1R: insulin‐like growth factor 1 receptor; FBXW7 and EFNA1: two endogenous endothelial genes; ↑: up‐regulation; ↓: down‐regulation

PMVs containing miR‐223 have been shown to regulate genes in HUVEC, including two endogenous endothelial genes: FBXW7 and EFNA1.^82^ In another experiment, platelet‐secreted miR‐223 via PMVs targeted endothelial cell insulin‐like growth factor 1 receptor (IGF‐1R) and thus promoted cell apoptosis induced by advanced glycation end products.^90^ In both experiments, platelet activation and MV release were induced upon incubation with thrombin, but the effects of MV containing miR‐223 on endothelium were different. The difference between the two experiments is that only the first experiment emphasized the combination of miR‐223 and AGO2. Perhaps this just shows the characteristic of miRNA regulating multiple target genes, but we cannot exclude the influence of AGO2 on the effect of MVs and their associated miRNA on endothelial function. Altogether, these findings suggest a specific role of PMVs containing specific miRNAs in vascular endothelial dysfunction (Figure 3).

### 4.2 Endothelial cell‐derived microvesicles and their miRNA cargo

Circulating endothelial cell‐derived microvesicles (EMVs) act as an important marker of vascular function and major adverse cardiovascular events in patients with endothelial dysfunction.^91^, ^92^, ^93^ Interestingly, EMVs have additional biological carrier function, which may provide different explanation for their role in pathology.^94^, ^95^ For instance, Hergenreider et al showed that endothelial‐derived miR‐143/145 could be transferred to SMCs via EMVs and then prevented SMC dedifferentiation to prevent atherogenesis.^96^

To illustrate molecular mechanism of EMVs containing miR‐19b in atherosclerosis, two studies demonstrated that EMVs with a high level of miR‐19b could inhibit endothelial cell migration and angiogenesis by targeting Rho GTPase‐activating protein 5 (ARHGAP5) and transforming growth factor β2 (TGFβ2), which modulate the function of HUVECs.^97^, ^98^ During atherosclerosis, miR‐126, which is selectively enriched in EMVs from apoptotic endothelial cells, suppresses the inhibitory function of regulator of G‐protein signalling (RGS16) and thus unleashes CXCR4 (CXC chemokine receptor type 4) to trigger a self‐amplifying feedback loop, which leads to increased production and release of atheroprotective chemokine CXCL12.^99^ In 2014, Schober et al first described that miR‐126‐5p preserved EC proliferation after hyperlipidaemic stress and protected from atherosclerosis by suppressing Notch1 inhibitor delta‐like 1 homolog (Dlk1), but endogenous miR‐126‐3p had no such effect.^52^ However, the same group later found that application of exogenous miR‐126‐3p contained by EMVs or apoptotic bodies promoted endothelial repair and inhibited atherosclerosis.^99^, ^100^ Therefore, we can speculate that the function of miRNAs depends on its endogenous expression or exogenous application, and even the same type of miRNA may have different effects.

In 2013, Jansen et al revealed that miR‐126 in EMVs released from apoptotic ECs inhibited the target protein sprouty‐related, EVH1 domain‐containing protein 1 (SPRED1) expression, and then promoted endothelial target cell migration and proliferation in vitro and reendothelialization in vivo to promote endothelial repair. Mechanisms by which SPRED1 inhibits Ras/MAPK (mitogen‐activated protein kinase) signalling to reduce migration and proliferation of ECs may be involved in this process. Interestingly, glucose‐damaged EMVs contained significantly lower amounts of miR‐126 and showed reduced endothelial repair capacity.^55^

**Figure 4:**
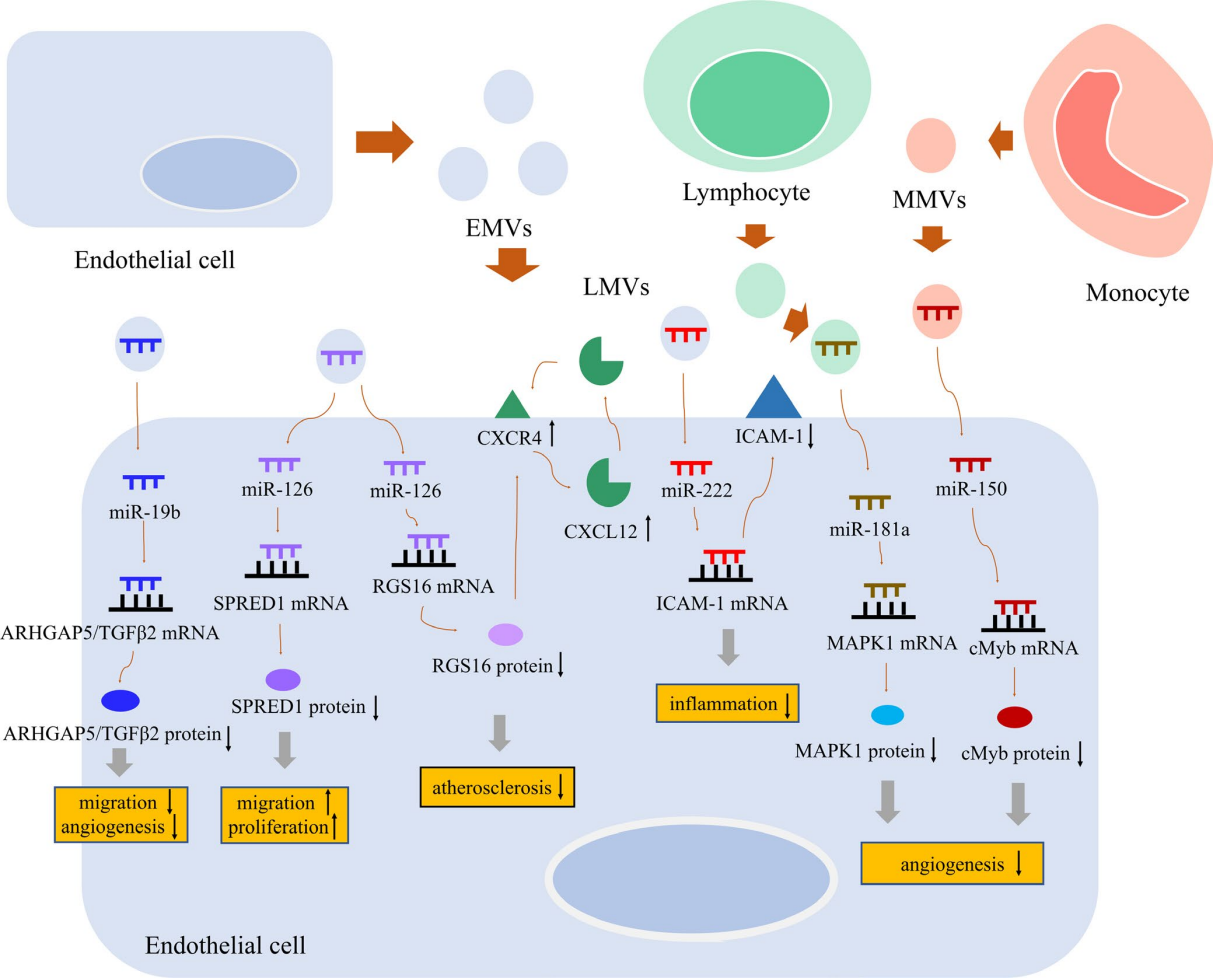
The role of EMVs, LMVs and MMVs containing specific miRNAs in vascular endothelial dysfunction. Endothelial cells, lymphocytes and monocytes produce microvesicles under stimulation. miRNAs contained in EMVs, LMVs and MMVs regulate the expression of target genes to affect the function of vascular endothelial cells, including cell migration, proliferation and inflammation. EMVs: endothelial cell‐derived microvesicles; LMVs: lymphocyte‐derived microvesicles; MMVs: monocyte‐derived microvesicles; ARHGAP5: Rho GTPase‐activating protein; TGFβ2: transforming growth factor beta2; SPRED1: sprout‐related, EVH1 domain‐containing protein 1; RGS16: regulator of G‐protein signalling; CXCL12: chemokine; CXCR4: CXC chemokine receptor type 4; MAPK1: mitogen‐activated protein kinase 1; cMyb: transcription factor; ↑: up‐regulation; ↓: down‐regulation

In 2015, Jansen et al demonstrated that miR‐222 in EMVs functionally reduced expression of its target protein ICAM‐1 and then promoted anti‐inflammatory effects in ECs, while miR‐222 in EMVs derived from glucose‐treated ECs facilitated ICAM‐1 and VCAM‐1 expression in resting ECs. Following experiment found that under pro‐inflammatory condition, miR‐222 in EMVs derived from glucose‐treated ECs rather reduced ICAM‐1 expression in target ECs.^94^ The contradictory results show that the effects of MVs and their miRNA cargo may depend not only on the state of the parent cells but also on the state of recipient cells. In summary, EMVs containing miRNAs are involved in the regulation of endothelial function through different mechanisms (Figure 4).

### 4.3 Leucocyte‐derived microvesicles and their miRNA cargo

Lymphocyte‐derived microvesicles (LMVs) have been shown to have a strong inhibitory effect on proliferation of ECs.^101^, ^102^, ^103^ Inhibition of miR‐181a, which is one of the most abundant miRNAs in LMVs, significantly attenuates the effect of LMVs on EC proliferation. Because overexpression of miR‐181a strongly inhibited MAPK1 expression in ECs, Yang C et al speculated the anti‐angiogenic effect of miR‐181a because of interference with the MAPK1/VEGF signalling.^104^ However, the contradictory role of miR‐181a in modulating angiogenesis has been reported. These controversial findings may suggest that the role of miR‐181a depends on specific cell or tissue. It can also be speculated that the effect of miRNAs in MVs may also be cell‐ or tissue‐specific.

Transcription factor cMyb is responsible for the increased migratory capability of ECs. miR‐150 is contained in monocyte‐derived microvesicles (MMVs), which can be transferred by MMVs into ECs to take part in down‐regulating cMyb. In patients with severe atherosclerosis, MMVs are rich in miR‐150; thus, the MMVs containing miR‐150 might be part of the reason for the vascularization of atherosclerotic plaques (Figure 4).^105^

### 4.4 Tumour cell‐derived MVs and their miRNA cargo

Tumour cells have been shown to produce large numbers of MVs. Some studies demonstrated that tumour cell‐derived MVs could regulate EC function.^106^

Angiogenesis plays a crucial role during tumorigenesis.^107^ Zhuang et al described that five tumour cell lines induced up‐regulation of miRNAs in EC and modified EC function through MV‐derived miRNAs.^108^ Furthermore, they described that miR‐9 was transferred into ECs by MVs and then activated the JAK/STAT pathway by down‐regulating suppressor of cytokine signalling 5 (SOCS5) to induce EC migration.

### 4.5 Stem cell‐derived MVs and their miRNA cargo

Human bone marrow‐derived mesenchymal stem cells (MSCs) and liver resident stem cells (HLSCs) have been shown to release MVs to transfer miRNAs to target cells, while the biological effect of stem cells may depend in part on MV‐shuttled miRNAs.^62^

RNA analysis revealed that miRNAs were enriched in adipose‐derived stem cells (ASCs) released MVs, and an underlying mechanism of the pro‐angiogenesis may be the delivery of miR‐31 via MVs from ASCs to ECs. miR‐31 in MVs from ASCs contributed to the migration and tube formation of ECs by targeting and suppressing factor‐inhibiting HIF‐1.^109^

## 5 REGULATION OF MICRORNAS SORTING INTO MVs

In 2013, Villarroya‐Beltri et al demonstrated the RNA‐binding protein heterogeneous nuclear ribonucleoprotein A2B1 (hnRNPA2B1) as a key player in miRNA sorting and loading into exosome through combining with GGAG motifs on miRNA.^110^ Recently, some studies found that RNA‐binding proteins hnRNPQ and hnRNPU were also involved in exosomal miRNA sorting.^111^, ^112^ Besides, Ago2, the Y‐box protein 1, ceramide signalling and mRNA‐miRNA interaction also play a role in regulating miRNA packing into exosome.^113^, ^114^, ^115^, ^116^ However, little is known about the mechanism by which miRNAs are selectively packaged into MVs. The process of sorting and export of miRNAs is ATP‐dependent and is affected by extracellular condition.^117^ We speculate that the content and mode of miRNAs packaging into MVs may depend on the nature of the agonist, stimulatory or shear conditions.^69^, ^82^

As far as we know now, firstly, inflammatory factors may be involved in regulating the level and transfer of miRNAs in MVs. Pan Y et al demonstrated that platelets contained abundant miRNAs, particularly miR‐223, and the level of the miRNAs in PMVs was up‐regulated by inflammatory factors such as thrombopoietin (TPO) and thrombin.^90^ Besides, Alexy T et al showed that pro‐inflammatory cytokine TNF‐α altered the release and transfer of miRNAs in EMVs.^71^ Codagnone M et al indicated that anti‐inflammatory pro‐resolution lipid mediator lipoxin A4 (LXA4) regulated the miRNAs in EMVs released by TNF‐α‐stimulated HUVECs.^68^ Kuhn S et al provided first evidence that anti‐inflammatory adenosine significantly increased miR‐142‐3p levels in MMVs.^67^ Furthermore, a study found miR‐221, miR‐320a, miR‐92a and miR‐17 were significantly higher (greater than twofold) in hydrochloric acid‐induced MV release from lung epithelium.^66^ Secondly, in addition to inflammatory factors, other factors can also regulate the transfer of miRNA in MVs. Collino F et al demonstrated that ribonucleoproteins (T‐cell internal antigen‐1 (TIA), TIA‐1‐related (TIAR) and AU‐rich element‐binding protein (HuR)) in MVs were involved in the selected pattern of miRNAs in MVs.^62^

In addition, packing of miRNAs into specific MVs may also be affected by diseases.^118^, ^119^ For instance, miR‐222 was demonstrated to be transported into recipient ECs by EMVs and functionally regulated expression of ICAM‐1. However, after simulating diabetic conditions, the data showed reduction in the number of miR‐222 in EMVs and reduced capacity of anti‐inflammatory in vitro and in vivo.^94^ It is speculated that the ‘packaging’ also depends on nucleases presenting in the recipient cell, proteins found in MVs and sex‐related differences (Table 1).^120^, ^121^ Although research on MVs has been ongoing for years, the determinants and regulatory factors of miRNA sorting and packaging into MVs need further research.^122^

**Table 1:** Factors that regulate miRNAs in MVs

miRNA	Experiment	Factor	Effect	MVs’ origin	Sample	Related diseases	Ref.
miR‐223	In vitro	1 ng/mL TPO or 0.1 U/mL and 1 U/mL thrombin	Up‐regulate	Human platelet	Venous blood	Enteritis, hepatitis, nephritis, atherosclerosis	90
miR‐221, 320a, 92a, −17	In vivo	Hydrochloric acid (0.1 N, pH 1.5)	Up‐regulate	Lung epithelial cell of mouse	Bronchoalveolar lavage fluid	Acute lung injury	66
miR‐126, −21, −155	In vitro	TNF‐α (100 ng/mL	70%‐80% decrease in miR‐126 and −21; a significant increase in pre‐miR‐155 and miR‐155; 50% reduction in uptake by recipient cells	Human aortic endothelial cells	‐	‐	71
miR‐181a,‐660,‐20b,‐29b,‐217,‐29a,‐100,‐92a,‐214,‐139,‐494,‐19a,‐19b,‐216,‐143,‐362,‐20a,‐126‐5p	In vitro	LXA4 (0.1‐100 nmol/L)	Up‐regulate miR‐126‐5p and down‐regulate the rest of 18 miR	Human umbilical vein endothelial cell	Umbilical cord of human	‐	68
miR‐125a, −34a	In vitro	Sex	miR‐125a was lower in activation‐derived EMVs, whereas expression of miR‐34a was higher in apoptosis‐derived EMVs from men compared with women.	Human endothelial cell	Venous blood of human	‐	120
miR‐142‐3p	In vitro	Adenosine (100 μmol/L)	Twofold increase in the miR‐142‐3p level in MMVs	Bone marrow mononuclear cell	Bone marrow from hind legs of mice	‐	67

Note

We listed the factors affecting miRNAs and some basic information in the experiments, as well as the diseases involved in these miRNAs changes. qRT‐PCR: quantitative real‐time polymerase chain reaction. TPO: thrombopoietin; TNF‐α: tumor necrosis factor‐α; LXA4: lipoxin A4.

## 6 EXISTING PROBLEMS OF MVs AND THE CLINICAL PROSPECTS

### 6.1 The present existing problems of MVs

Although the presence of MVs has been known for many years, many basic questions of MVs remain. Firstly, current isolation procedures do not clearly purify specific population of MVs, and this may partly be explained by the lack of standardization of both isolation techniques and protocols, such as specific markers of different MV populations.^123^, ^124^ Furthermore, MV counts, types and contents tend to vary per collection methods, storage media and the assay itself, which may also affect the type and quantity of miRNAs in MVs.^125^ Full exploitation of the information encompassed within blood MVs will require complicated proteomic, lipidomic, transcriptomic and metabolomic approaches.^126^ Secondly, it is not clear how specific miRNAs are packaged into MVs and transferred into target cells in response to different stimuli and pathological conditions. In the same way, the process of how MV‐delivered miRNAs are selectively internalized by the specific cells or tissues needs further research.^127^ Thirdly, although there is a specific effect of miRNAs on MVs, we cannot rule out the influence of other miRNAs or bioactive molecules in the MVs.^94^ Fourthly, both MVs and exosomes are surrounded by a phospholipid bilayer and carry RNAs and proteins,^128^ but we do not know whether there are differences in the transmission and function between MV‐mediated miRNAs and exosome‐mediated miRNAs.

Finally, in terms of treatment, because of the complexity and diversity of miRNA‐mRNA interaction, we need to take this into account that the impact of MV‐delivered miRNAs on the target cells may be greatly extensive to have bad effects. Besides, before using detections of the MV‐delivered miRNAs to better assess diseases, it is necessary to clarify the aspects of the clinical utility of MV as biomarker, such as evidence of their predictive value, discrimination and reclassification power.^26^ If all these outstanding issues can be resolved, the use of MV‐delivered miRNAs will be an effective and site‐specific treatment.

### 6.2 The clinical prospect of MVs

On the one hand, extracellular miRNAs hold great potential to act as disease biomarkers due to their non‐invasive accessibility and remarkable stability.^60^, ^77^, ^129^ A great number of studies have shown that miRNAs are differently enriched in MVs, and their expression patterns also change with respect to different diseases.^130^ Therefore, MVs and associated miRNAs are considered to be potential diagnostic biomarkers of diseases.^131^, ^132^, ^133^ For instance, in a prospective study, in 176 patients with stable coronary artery disease (CAD), Jansen F at al. found that the level of miR‐126 or miR‐199a expression in circulating MVs but not in plasma could predict the occurrence of cardiovascular events in stable CAD patients.^134^ Future studies must identify altered miRNAs in specific subclasses and demonstrate representative ranges of health and disease.^60^

On the other hand, MVs and the miRNAs they contain have inspired many studies on pathology and disease resistance.^58^, ^135^ For a long time, it has been thought that the therapeutic effect of MV‐delivered miRNAs is obviously superior to that of traditional treatment. Firstly, acting as therapeutic delivery agents, MVs originate from the host; thus, they have reduced toxicity and may be tolerated by the immune system.^136^ Secondly, because of the complexity and diversity of miRNA‐mRNA interaction, the impact of MV‐delivered miRNAs on the target cells may be quite extensive, potentially avoiding the undesired effects caused by switching a single target gene on or off.^137^ Many studies have shown that MVs delivering miRNAs are involved in the occurrence and development of many diseases.^131^, ^138^, ^139^ As a strategy to alter the type and quantity of extracellular miRNAs to gain therapeutic advantage, specific miRNAs or their inhibitors can be added to specific MVs.^140^ Future studies may focus on tailored recombinant MVs with unique cassettes of miRNAs for therapeutic benefits.^141^

## CONFLICT OF INTEREST

The authors declare no conflict of interest.

## AUTHOR CONTRIBUTIONS

Qiang Zhang, Zeyu Shu and Jin Tan conceived, designed and drafted the manuscript. Yuyang Miao and Zeyu Shu contributed to the revised version of the manuscript. All authors confirmed the final version of the manuscript for submission.

## Data Availability

I confirm that I have included a citation for available data in my references section.
